# A novel insertion mutation identified in exon 10 of the *MEFV* gene associated with Familial Mediterranean Fever

**DOI:** 10.1186/1471-2350-15-74

**Published:** 2014-07-01

**Authors:** Hasan Dogan, Fatih Akdemir, Sener Tasdemir, Omer Atis, Eda Diyarbakir, Rahsan Yildirim, Mucahit Emet, Mevlit Ikbal

**Affiliations:** 1 Department of Medical Biology, Medicine Faculty, Ataturk University, 25240 Erzurum, Turkey; 2 Department of Medical Genetics, Medicine Faculty, Ataturk University, 25240 Erzurum, Turkey; 3 Department of Internal Medicine, Medicine Faculty, Ataturk University, 25240 Erzurum, Turkey; 4 Department of Emergency Medicine, Medicine Faculty, Ataturk University, 25240 Erzurum, Turkey; 5 Department of Medical Genetics, Medicine Faculty, Karadeniz Technical University, 61080 Trabzon, Turkey

**Keywords:** DNA sequencing, FMF, *MEFV*, Novel mutation, Exon 2, Exon 10

## Abstract

**Background:**

Familial Mediterranean Fever (FMF), characterized by recurrent fever and inflammation of serous membranes, is an autosomal recessive disease caused by mutations in the Mediterranean fever (MEFV) gene. Around 296 mutations have been reported to date.

**Methods:**

Two two-generation Turkish families with a total of four members diagnosed with FMF clinically were screened with DNA sequencing performed on exon 2 and exon 10 of the MEFV genes. Then, complete exome sequencing analysis of *MEFV* gene was done for four patients in whom novel mutation was detected.

**Results:**

A novel single base Guanine (G) insertion mutation in the coding region of MEFV gene, named c.2330dupG (p.Gln778Serfs*4 or Q778SfsX4) resulting in a mutated Pyrin/Marenostrin protein was identified.

**Conclusions:**

This is the first report of a new mutation in exon 10 of the MEFV gene in two Turkish families. This novel pattern of insertion mutation may provide important information for further studies on FMF pathogenesis.

## Background

Familial Mediterranean Fever (FMF) is a hereditary auto inflammatory disease, predominantly affecting people of Mediterranean and Middle Eastern descent, especially Jews, Turks, Armenians, Greeks and Arabs. It is characterized by recurrent attacks of fever, abdominal pain with peritonitis, pleuritis, arthritis, skin lesions (erysipelas-like erythema) and renal amyloidosis [1,2]. Systemic amyloid A (SAA) protein deposition especially in the kidneys makes FMF a potentially fatal disease [1]. Since clinical diagnosis is difficult, identification of disease causing mutations is crucial [2].

The FMF gene is the Mediterranean FeVer *(MEFV)* gene, comprises 10 exons, is located on the short arm of chromosome 16 at position p13.3 and encodes for the 781 amino-acid protein named as pyrin or marenostrin [2-4]. Disease-associated mutations have been identified; comprised approximately 85% of the mutations in exon 10 and exon 2 [5]. To date, more than 296 gene mutations and polymorphisms have been discovered in the gene [6]. Still new mutations and their association with FMF are understudied. Among these mutations, there is only one insertion mutation on exon 2 that was reported in 2002 [6].

Generally FMF is inherited in an autosomal recessive manner, but recent studies have showed that some cases that are heterozygotes can manifest a clinical symptom consistently with mild FMF [7].

In this study, four patients with FMF symptoms who have two ancestries with a single base Guanine (G) insertion/duplication mutation on exon 10 were showed.

## Methods

### Subjects

Each year, approximately 1200 clinically pre-diagnosed FMF cases are referred to our laboratory by their physicians to detect *MEFV* mutations. Among those patients with clinical evidence for FMF, 4 individuals from two Eastern Anatolian Turkish families were studied. This study approved by the local institutional review board and ethics committee of Ataturk University, Medical Faculty, Erzurum, Turkey (no. B.30.2.ATA.0.01.00/63). Informed consent for the publication of this data was obtained from participants or their parents for the individuals under 18 years old.

### PCR amplification and sequencing

From each patient, 2 cc peripheral venous blood specimens were collected in test tubes containing anti-coagulant (EDTA). Genomic DNA was isolated with an automatized DNA isolation device (MagNA Pure LC DNA Isolation Kit I, Roche) according to manufacturer’s instructions.

Exon 2 and exon 10 was sequenced by using GML SeqFinder Sequencing System *MEFV* kit and confirmation sequenced was done by the following primers Exon 2: F: 5′-CTAAACGTGGGACAGCTTCATC-3′ and R: 5′-CTTCCTTCAGGTCCGCAGAT-3′; Exon 10: F: 5′-GACTTGGAAACAAGTGGGAGAG-3′, and R: 5′-CAGGAAGAGAGATGCAGTGTTG-3′ using Sanger sequence technology. PCR conditions were as follows: Initial denaturation at 94°C for 5 minutes, 35 cycles at 94°C for 30 seconds and 58°C for 45 seconds, 72°C for 1 minute, and a final extension at 72°C for 5 minutes. The PCR products were visualized and collected with 2% agarose gel electrophoresis followed by purification using Exo-SAP PCR purification Kit (UAB Corporation, Cleveland, Ohio, USA) The product was sequenced in both strands initiating from the forward and the reverse primers used in the initial PCR and analysed on an ABI 3130 Automated DNA Sequencer (Applied Biosystems, Foster City, CA, USA). Further analysis was performed with SeqScape v2.6 and Sequencing Analysis 5.3.1 version programs.

We checked the whole coding region to search for a mutation on the second *MEFV* allele although there were 4 individuals with novel *MEFV* mutation in the heterozygous state. Whole exome MEFV gene sequencing of four patients in whom novel mutation was performed with the 454 GS-Junior Next Generation Sequencer platform (Roche Diagnostics, Mannheim, Germany), according to manufacturers’ instructions. Analysis was performed with JSI Medical Systems in SeqNext Software (Roche Diagnostics, Mannheim, Germany) programs.

In both sequence analyses, the reference coding sequence of the wild-type *MEFV* was obtained from the NCBI, Gene Bank accession number AF111163 (FMF; OMIM no. *249100) (http://www.ncbi.nlm.nih.gov/).

## Results

**Figure 1:**
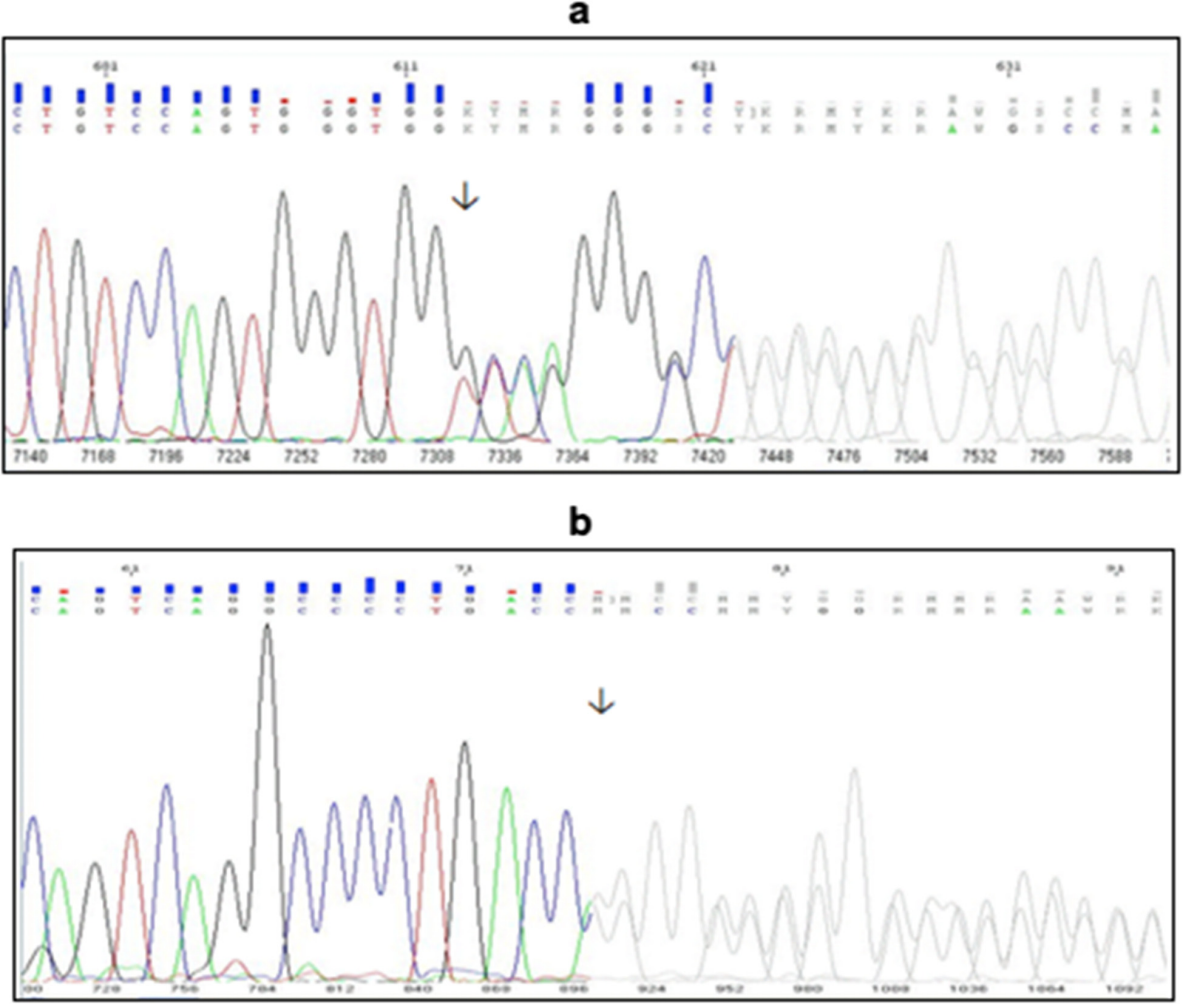
**Electrophoregrams of the new heterozygous insertion at 2331**^**st **^**nucleotide of *MEFV*.** The insertion resulted frame shift in codon 777. **(a)** Forward Electrophoregram, **(b)** Reverse Electrophoregram.

**Figure 2:**
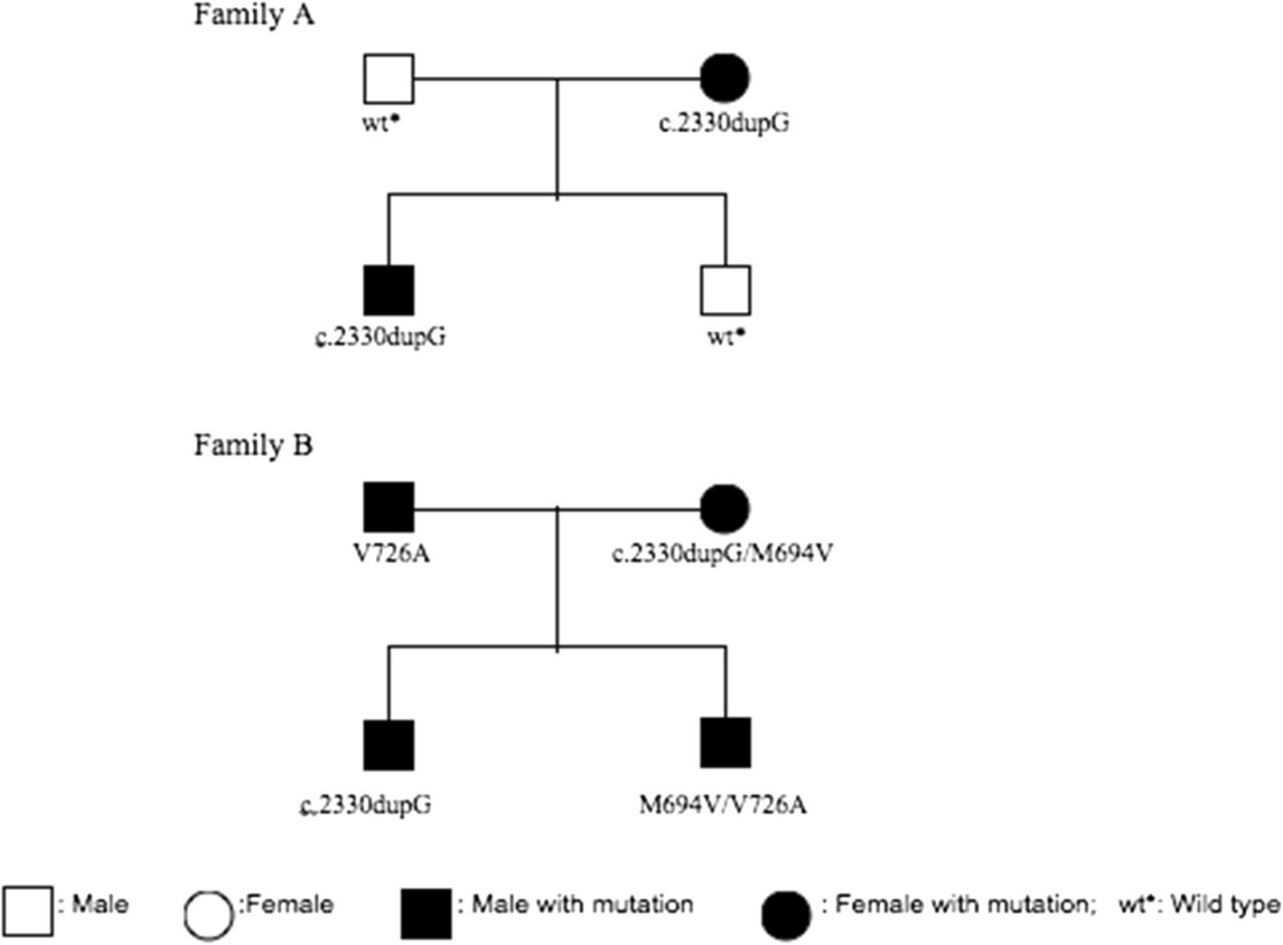
Pedigree diagrams of the probands families.

Two unrelated patients aged 16 and 7, who were clinically suspected of FMF with mild symptoms of recurrent attacks of fever (1-3 days) and abdominal pain were referred separately to our laboratory by their physicians for routine analysis of *MEFV* mutation detection. In both patients a heterozygous single G nucleotide duplication resulting in frame shift mutation in exon 10 at 777^th^ codon, (between 2330^th^ and 2331^st^ nucleotides) was detected (Figure 1). The frame shift mutation was confirmed by sequencing in both directions. Next, DNA samples from first-degree relatives of the two patients (4 individuals from each family, 8 subjects totally) were collected to screen for mutations at exon 2 and exon 10. Although no variations were detected by whole exome sequencing of all 10 exons of *MEFV* gene in 4 individuals from each family, the mothers of both patients have the same heterozygous frame shift mutation as the patients (Table 1). The pedigrees of the families and the mutations that were found are shown in Figure 2. Due to the G nucleotide insertion and frame shift mutation between 2330^th^ and 2331^st^, the nucleotide sequence converted from ggt cag ggg cct gac tga to ggG tca ggg gcc tga and this conversion resulted in a stop at codon 780. The protein sequence converted from Gly-Gln-Gly-Pro-Asp-STOP to Gly-Ser-Gly-Ala-STOP (p.Gln778Serfs*4 or Q778SfsX4).

**Table 1:** Clinical symptoms and results of mutation analysis of the two families

**Family**	**Relation**	**Gender, age (year)**	**Age of symptoms onset**	**Exon 10 mutation type**	**Common FMF symptoms**					
					**Abd. pain**	**Fever**	**Chest pain**	**Arthritis**	**Skin lesions**	**Amyloidosis**
Family A	Father	M, 41y	No Sx*	wt**	-	-	-	-	-	-
	Mother	F, 35y	30y	c.2330dupG/0	+	+	-	-	-	-
	Child 1^1^	M, 16y	15y	c.2330dupG/0	+	+	-	+	-	-
	Child 2	F, 10y	No Sx	wt**	-	-	-	-	-	-
Family B	Father	M, 35y	No Sx	V726A/0	-	-	-	-	-	-
	Mother	F, 32y	20y	c.2330dupG/M694V	+	+	+	-	-	-
	Child 1^1^	M, 7y	6y	c.2330dupG/0	+	+	-	-	-	-
	Child 2	M, 10y	3y	M694V/V726A	+	+	-	-	-	-

No Sx*: No Symptoms; wt**: Wild type; ^1^: Probands.

On six different mutation analyser web-based tools, c.2330dupG mutation was searched. Align GVGD and PolyPhen-2 (HumDiv Prediction version) showed c.2330dupG mutation might have pathogenic effect (Table 2) [8-13].

**Table 2:** Results of mutation analyser web-based tools

	**Computational pathogenicity predictions**			
	**Prediction**	**Score 1**	**Score 2**	**Score 1&2 description**
http://agvgd.iarc.fr/agvgd_input.php[8,9]	Class C65	0.0	68.20	(GV, GD) Prediction ranges from C0-benign to C65- pathogenic
http://genetics.bwh.harvard.edu/pph2/ (HumVar Prediction) [10]	Benign	0.265		
http://genetics.bwh.harvard.edu/pph2/ (HumDiv Prediction) [10]	Possibly damaging	0.876		
http://sift.jcvi.org/[11]	Tolerated	0.1	3. 08	SIFT Score. Ranges from 0 to 1. The amino acid substitution is predicted damaging is the score is < = 0.05, and tolerated if the score is > 0.05.
http://provean.jcvi.org/seq_submit.php[12]	Neutral	-1.027		
http://www.mutationtaster.org/[13]	Polymorphism			

The patients did not fulfil the major criteria of FMF disease, although they both showed mild symptoms of minor criteria (recurrent abdominal pain and recurrent attacks of fever) (Table 1). Detailed information about the two patients and their families is as follows:

### Family A

16-year-old male (Child 1) with mild FMF symptoms and arthritis. c.2330dupG mutation was detected with DNA sequencing. After screening the family, the mother; whose suffered mild symptoms of recurrent attacks of fever (1-3 days) and abdominal pain also, was found to have the same insertion as patient A.

### Family B

7-year-old male (Child 1) with mild FMF symptoms having a sibling diagnosed with FMF and an asymptomatic father carrying the heterozygous common mutation V726A. Because the family has another child with FMF and they know that they are carriers, child 1 was admitted by us early for genetic testing. The mother is a carrier of M694V, showing the symptoms of chest pain, recurrent abdominal pain and recurrent fever. Upon testing by DNA sequencing, heterozygous c.2330dupG was detected in Child 1. Also his mother has been detected as compound heterozygous due to both M694V and c.2330dupG mutations.

## Discussion

**Figure 3:**
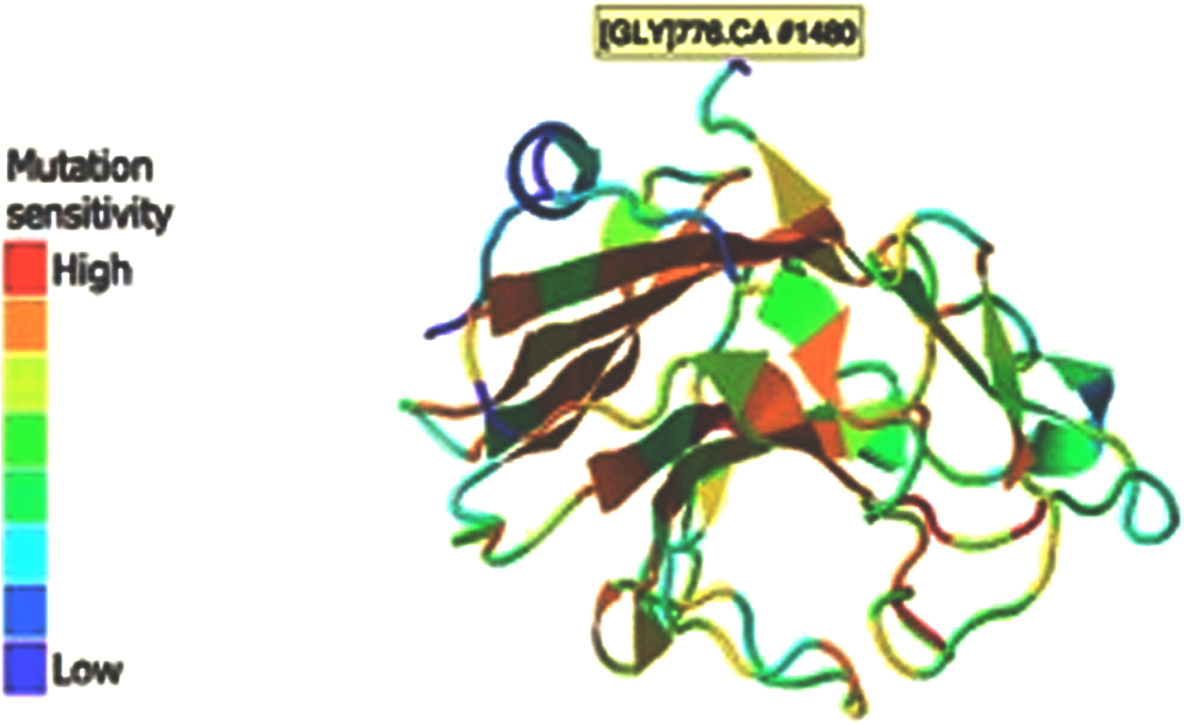
**3D view of pyrin protein **[18]**.**

In this study, we report a novel frame shift mutation of the *MEFV* gene; c.2330dupG that was detected in 4 FMF patients in 2 families from eastern Turkey. The supporting findings which analysed from different web-based online tools, and outcomes from the patients whom are subject of this article whether this novel mutation causes FMF and may have genotype/phenotype correlations are as follows: Firstly, c.2330dupG mutation was found in patients with clinical symptoms characteristic for FMF and it has not been found in any of the subject *MEFV* genes from unaffected Turkish inhabitants so far among the regional 2,530 referrals, 1,048 cases (41.42%) of them had the known mutations of *MEFV*[14]. Secondly, the mother of Family B is a carrier with M694V common mutation, which is one of the best-characterized mutations known to cause FMF in a recessive manner. In addition to this, she is also heterozygous for c.2330dupG mutation and she also has mild FMF symptoms. Therefore, we think that the insertion mutation probably caused FMF disease by compound heterozygousity. Thirdly, this novel mutation was found in exon 10 of *MEFV*, where most mutations that are identified as FMF causing mutations are located. The structure of the C-terminal domain has been described, although this very C-terminal region in which our insertion resides does not have a definitive structure [15]. The C-terminal B30.2 domain is also named the PRYSPRY domain and has the functions of ligand binding, signal transduction and mediating protein–protein interactions. The mutations may diminish or eliminate the ability of pyrin, consequently suppressing apoptosis and IL-1B activation [16,17]. The novel mutation is very near to the end of the C-terminal, after the B30.2 domain and there are 5 amino acids after this position. We made a model of this area of pyrin protein in 3D diagrams using web-based protein structure prediction databases, but the exact effect of this area is not understood (Figure 3) [18]. So far 3 different missense mutations have been described in this C-terminal region, although only one of them was reported as symptomatic [6]. Mutations at this location have potential roles on the function this protein.

Recent findings indicate the clinical disease with heterozygous mutations of *MEFV* gene and thus these mutations may act as dominant negative mutations or pseudo-dominantly (Manifesting heterozygousity FMF; OMIM no. *134610) [7,19,20]. Although the mother in family A and two probands are associated with mild FMF symptoms, they are heterozygous. Therefore, the insertion mutation presented in this article may cause FMF disease by dominant inheritance. Further molecular biology studies should have been done to proof this. The ratio of patients with a single mutation varies between 16.5% and 33.8% for FMF. These mutations clinically caused mild inflammatory process [21,22]. Marek-Yagel et al. [20] and van Gijn et al. [23] tried to discover a second mutation on the second allele in the *MEFV* gene of heterozygous patients by investigating the coding region, the exon and intron boundaries, as well as in the promoter region of the gene. Although they performed complete sequencing of the gene, they were unable to detect a second mutation. Later, it became obvious that FMF is not fully recessive and that in some cases heterozygous mutations are associated with mild clinical symptoms. A complete sequencing of all 10 exons of the MEFV gene in four individuals’ carriers of a novel mutation did not show the presence of a second pathogenic mutation. We can therefore conclude that the symptomatic two probands and the mother of family A are true heterozygous and the mother of family B is compound heterozygous as she has got both M694V and c.2330dupG mutations. Based on these findings and our own, we can say that the simple heterozygous insertion mutation can cause FMF disease.

The novel mutation was present in a heterozygous state in four subjects, located in exon 10 of the gene and causes amino acid replacement after the 777^th^ amino acids. This mutation caused a new amino acid sequence that is G777G, Q778S, G779G, P780A and D781stop; and shortened one amino acid. This novel frame shift mutation is found in exon 10 of *MEFV* gene, where previously identified FMF causing mutations are located.

A frame shift mutation involves the insertion or deletion of a nucleotide in which the number of deleted base pairs that is not a multiple of three and consequently disrupt the triplet reading frame, generally leading to the formation of premature termination (STOP codon) and resulting in a truncated protein product. To date for FMF two ‘in frame’ deletion mutations have been described, I692del and M694del. So far, no insertion has been reported at exon 10. At exon 2, four insertions have been reported, 334_335insG (p.Glu112fs*130), 390_391insGAGGGGAAC (p.Glu128_Asn130dup), 606_621dup (p.Ser208Alafs*39) and 761_764dupCCGC (p.Asn256Argfs*70) [1,6,24]. Only one of these is a single G base insertion as it is in our cases. No frame shift mutations have been described at other exons [6]. The insertion described in this article is significant because it is the only insertion and frame shift mutation reported at exon 10 so far.

## Conclusions

In conclusion, to our knowledge, this mutation has not been reported before. Therefore, we think the c.2330dupG (p.Gln778Serfs*4) mutation should be listed in the *MEFV* sequence variants of FMF in databases. In order to identify specific phenotype/genotype correlations exactly, the novel mutation and its relationship should be confirmed by studying homozygous individuals for this mutation and by sequencing the *MEFV* gene completely to rule out other possible mutations.

## Abbreviations

MEFV: Mediterranean fever; FMF: Familial Mediterranean Fever; EDTA: Ethylenediaminetetraacetic acid; ABI: Applied biosystems; NCBI: National Center for Biotechnology Information; OMIM: Online Mendelian Inheritance in Man.

## Competing interests

The authors received no financial support for the research and/or authorship of this article. The authors declare that they have no competing interest to the publication of this article.

## Authors’ contributions

HD, Assistant Professor Doctor, design of the study, data analysis, edited manuscript. FA, Assistant Professor Doctor, edited manuscript. ST, Assistant Professor Doctor, consulted the subjects. OA, Assistant Professor Doctor, acquisition of data. ED, Assistant Professor Doctor, analysed data. RY, Associate Professor Doctor, referred the subjects. ME, Associate Professor Doctor, edited manuscript. MI, Professor Doctor, design of the study. All authors read and approved the final manuscript.

## Pre-publication history

The pre-publication history for this paper can be accessed here:

http://www.biomedcentral.com/1471-2350/15/74/prepub
